# Traditional menstruation practices among Nepalese women living in Japan

**DOI:** 10.1186/s12905-022-01789-9

**Published:** 2022-05-31

**Authors:** Rina Kawata, Masayuki Endo, Kazutomo Ohashi

**Affiliations:** 1 Division of Health Science, Graduate School of Medicine, Osaka University, 1-7, Suita, Osaka, 565-0871 Japan; 2 Faculty of Global Nursing, Otemae University, 2-1-88, Cyuoku Otemae, Osaka, 540-0008 Japan

**Keywords:** Traditional menstruation practices, Nepal, Japan, Immigrants, Reproductive health, Menstrual hygiene management

## Abstract

**Background:**

Traditional menstruation practices in Nepal (*Chhaupadi* in Nepalese) lack scientific support and undermine women’s health. This study aimed to understand the changes in the traditional menstruation practices due to migration from Nepal to Japan.

**Methods:**

This study included 104 Nepalese women of reproductive age living in an urban area of western Japan. Participants were recruited using snowball sampling, and the method of data collection was a questionnaire survey. To examine how Nepalese women adapt traditional menstruation practices to their living environment, we compared how women followed frequently 17 traditional practices when they lived in Nepal and later in Japan. We examined the relationships among behavioural changes in traditional practices, education level, and caste.

**Results:**

The frequency of 14 of the 17 traditional practices decreased after the women moved to Japan. Among women who reduced the frequency of traditional practices after moving from Nepal to Japan, the reduction was not associated with educational level or caste of the participants.

**Conclusions:**

This study suggests that the attitudes towards the traditional menstruation practices change in response to living circumstances. Future studies should focus on determining living environment factors related to behavioural changes in traditional practices.

## Background

No supporting scientific evidence exists for traditional menstruation practices observed in Nepal (*Chhaupadi* in Nepalese); menstrual blood is not pure in Nepalese society, and hence, women are required to isolate during menstruation. Consequently, they are compelled to live in an outdoor hut and prohibited from entering public areas, such as schools, temples, and places supplying drinking water. They are allowed to touch others and from entering the kitchen or bedroom for the duration of their menstruation. They cannot share water or food with others, and not consume nutritious foods, such as milk and other dairy products, meat, fruits, and green vegetables [1, 2], and often survive only on rice, salt, and cereals/dry foods [2]. These traditional practices are widely followed in both the rural and urban areas [3, 4].

The Ministry of Health in Nepal reported that women may develop anaemia due to their poor nutritional statuses when living in outdoor huts during their menstrual period [5]. Other research also suggests that children’s health also deteriorate during their mothers’ menstrual periods [1]. The Nepalese government has therefore taken measures to reduce traditional menstruation practices. In 2005, the Supreme Court of Nepal ordered the government to enact legislation to eliminate these traditional practices, and the Nepalese Provisional Charter enacted a law protecting the right to reproductive health in 2007 [6, 7]. In addition, the Ministry of Women, Children, and Social Welfare in Nepal established guidelines for the elimination of traditional menstruation practices in 2008 [7]. However, despite it being a criminal offence to force a woman to take part in these practices, the deaths of women and children due to traditional menstruation practices were reported in 2019 [8, 9]. Wild animals caused most deaths; the sexual victimization was not reported possibly due to social concerns and safeguarding the future of unmarried women [2, 7, 9].

The behavioural restrictions imposed by traditional menstrual practices in Nepal result in a lack of hygienic menstrual coping behaviour [1]. This has resulted into a trend with increased cases of dysmenorrhoea [10] and urinary tract infections [11]. Further, school absenteeism, possibly due to menstrual cramps, has become a social problem. The majority of women are not adequately educated about menstruation and how to cope with it, and feel confused, fearful, stressed, inconvenienced, and embarrassed by traditional practices related to menarche and menstruation at the time of first menstruation [1, 3]. Menstrual prejudices and practices hinder the opportunity to receive scientific knowledge about menstruation. This may have a negative impact on hygienic menstrual management and women's mental and physical health. Even in areas and communities in Nepal where outdoor segregation is less common, menstrual taboos such as not going to school or work or sleeping in bed during menstruation remain part of women's daily routines, which affect their lives [4]. Therefore, to protect women's health, urgent elimination of traditional menstruation practices from the society is required.

Crawford, Menger, and Kaufman reported that women living in urban areas in Nepal manage menstruation in both the traditional and modern ways [3]. Further, Nepalese women followed traditional menstrual practices for reasons of social acceptance, such as respect for their elders, or a desire to continue cultural traditions. However, Nepalese women tend to accept alternative and modern approaches to menstruation when prejudice against it is reduced in their communities and family members permit them to cease traditional menstruation practices [3]. Historically, the community castigates Nepalese women when they do not follow these traditional practices [8], and while most girls abide by them every month, they would prefer to discontinue the practices [11]. Based on these studies, we hypothesised that Nepalese women would change their attitudes towards traditional menstrual practices if they lived outside Nepal and were not forced to follow the tradition by their communities.

To examine how Nepalese women adapted the traditional menstruation practices to their living environment, we compared the frequency of traditional practices with women living in Nepal and Japan, and examined the relationships between changes in the frequencies of practices based on education levels and castes. We also abstracted negative and positive attitudes about traditional practices related to menstruation from participants’ opinions. Based on the results, we suggest ways of reducing these traditional practices in Nepal.

## Methods

### Participants

This cross-sectional study was conducted between April 2016 and August 2016, and included Nepalese women, aged 20–45 years, living in Osaka and Hyogo prefectures in western Japan.

The study included a questionnaire, which evaluated demographic characteristics (present age, age when they started living in Japan, length of stay in Japan, religion, educational level, and caste) and the frequencies of traditional menstruation practices while living in Nepal and Japan. At the end of the questionnaire, we also asked the participants “Please write any comments you may feel regarding the difficulties or differences in menstrual practices between Nepal and Japan.”

Researchers of Osaka University extracted the descriptions of traditional menstruation practices in Nepal from previous studies [1–3, 12] and discussed them with two Nepalese individuals living in Japan (a research assistant and a translator). Traditional menstruation practices consisted of six categories and following five restrictions: (1) restriction of religious behaviours, (the inability to go to temples or attend religious events and weddings), (2) restriction of contact with others (the inability to interact with or attend to others, particularly male family members), (3) restriction of cooking and eating (the inability to cook, use the kitchen, use cookware, touch fruit or fruit trees, or eat nutritious food), (4) restriction of living place (the inability to live at home, use the bedroom, or go to public places and others’ homes), and (5) restriction of sanitary behaviours (the inability to shower or use new sanitary napkins).

Responses on the frequencies of traditional menstruation practices were scored using a 5-point Likert scale of 1 (*never*), 2 (*rarely*), 3 (*sometimes*), 4 (*often*), and 5 (*always*). A higher score indicated a higher frequency of following the traditional menstrual practices. In this study, we compared the frequencies of each participant when living in Nepal and Japan.

Furthermore, we asked participants about the difficulties or differences in menstrual practices when they were in Nepal and Japan. A Nepalese research assistant read the questionnaire and provided additional explanation in cases where the participant did not understand the questionnaire. After completing the questionnaire, a research assistant translated the answer from Nepali to Japanese.

### Preliminary examination

We first developed the questionnaire in Japanese and then translated it into Nepali. To evaluate the validity of the items of traditional menstruation practices, we asked eight Nepalese women living in Osaka to cooperate with a preliminary examination. A researcher (Japanese), two Nepalese research assistants, and eight participants discussed the questionnaire’s contents and, subsequently, revised it.

### Survey

Two Nepalese research assistants asked the participants to gather at meeting places in their communities to participate in the study. The participants were recruited through snowball sampling. We also used flyers and posters that included the contact addresses and telephone numbers of the research collaborators that could be contacted to participate in this study. The research assistants verbally explained the purpose of this study and requested cooperation using documents written in Nepali. Along with the Japanese researcher, the research assistants distributed questionnaires to those who consented to participate. The Japanese researcher immediately left the room to avoid any risk of women feeling pressured to participate. The questionnaire survey was an anonymous and self-administered. When participants could not sufficiently understand the contents of the questionnaire, the research assistants provided additional explanations. The participants placed the completed questionnaires into a collection box located in the room, and were collected by the Nepalese research assistants. The research assistants remained neutral and were careful not to induce the participants to answer.

### Analysis

We divided the participants into two groups according to the frequency of traditional menstruation practices: the high-frequency group with scores of 4 and 5 and the low-frequency group with scores of 1, 2 and 3. To examine the behavioural changes in traditional practices related to menstruation, McNemar’s test, a test to interrogate the paired binomial data and analyze differences before and after a particular treatment in a population [13], was used to examine the relationship between the frequencies of following the traditional menstruation practices in Nepal and Japan. Next, we focused on behavioural changes in the high-frequency group in Nepal and examined the relationships between frequencies and demographic characteristics (education level and caste), using Fisher’s exact test. Furthermore, we also categorized free comments with reference to the Nepalese women's attitudes toward traditional menstruation practices extracted from the qualitative research of the previous study [3]. We used SPSS for Windows version 26 (IBM, Tokyo, Japan) to analyse the data, and the significance level was set at 5%.

## Results

Table 1 shows the demographic characteristics of the study participants. The average age of the participants was 29.7 ± 6.0 years, while average age when the subjects started living in Japan was 26.0 ± 5.1 years, and average length of stay in Japan was 3.7 years (range 0–17 years). Among the 104 participants, 90.4% were Hindus, 69.2% had high school or higher education levels, and 67.3% belonged to higher castes (Brahmin and Chettri).

**Table 1:** Demographic characteristics of the study participants (n = 104)

Age (years); mean ± SD (range)	29.7 ± 6.0 (20–45)
Age started living in Japan (years); mean ± SD (range)	26.0 ± 5.1 (16–43)
Length of stay in Japan (years); mean ± SD (range)	3.7 ± 6.0 (0–17)
*Religion; n (%)*	
Hindu	94 (90.4)
Buddhist	9 (8.7)
Christian	1 (1.0)
*Education level, n (%)*	
High school or higher	72 (69.2)
Secondary school or lower	32 (30.8)
*Caste, n (%)*	
High (Brahmin and Chettri)	70 (67.3)
Others	34 (32.7)

Table 2 shows the frequencies of following the traditional menstruation practices while living in Nepal and Japan. For 14 of the 17 traditional practices, the number of women in the high-frequency group while living in Japan significantly decreased in comparison with that while living in Nepal. The highest percentages in the high-frequency group in Nepal were for the categories ‘Going to a temple’ (76.9%) and ‘Attending religious events’ (69.2%). Although these percentages decreased after moving to Japan, they remained relatively high (65.4% for ‘Going to a temple’ and 56.7% for ‘Attending religious events’).

**Table 2:** Implementation of traditional menstruation practices at high and low frequencies in Nepal and Japan (n = 104)

Traditional menstruation practices	Nepal		Japan		*p*^c^ n (%)	PD (95% CI)^d^	High in Nepal but low in Japan
	High^a^ n (%)	Low^b^ n (%)	High^a^ n (%)	Low^b^ n (%)			
*Restriction of religious behaviours*							
Going to a temple	80 (76.9)	24 (23.1)	68 (65.4)	36 (34.6)	0.031	0.12 (0.02–0.21)	19 (18.3)
Attending religious events	72 (69.2)	32 (30.8)	59 (56.7)	45 (43.3)	0.021	0.13 (0.03–0.22)	20 (19.2)
Attending a wedding ceremony	46 (44.2)	58 (55.8)	23 (22.1)	81 (77.9)	0.002	0.16 (0.07–0.26)	29 (27.9)
*Restriction of contact with others*							
Attending to male family members	39 (37.5)	65 (62.5)	18 (17.3)	86 (82.7)	< 0.001	0.20 (0.10–0.30)	27 (26.0)
Contact with male family members	34 (32.7)	70 (67.3)	16 (15.4)	88 (84.6)	0.001	0.17 (0.08–0.27)	23 (22.1)
Contact with others	27 (26.0)	77 (74.0)	12 (11.5)	92 (88.5)	0.007	0.14 (0.05–0.24)	21 (20.2)
*Restriction of cooking and eating*							
Using the kitchen	47 (45.2)	57 (54.8)	25 (24.0)	79 (76.0)	< 0.001	0.22 (0.11–0.33)	30 (28.3)
Cooking	46 (44.2)	58 (55.8)	23 (22.1)	81 (77.9)	< 0.001	0.21 (0.17–0.32)	29 (27.9)
Touching fruit and fruit trees	40 (38.5)	64 (61.5)	17 (16.3)	87 (83.7)	< 0.001	0.21 (0.12–0.30)	25 (24.0)
Using cookware	38 (36.5)	66 (63.5)	20 (19.2)	84 (80.8)	0.004	0.16 (0.06–0.26)	24 (23.1)
Eating nutritious food	14 (13.5)	90 (86.5)	7 (6.7)	97 (93.3)	0.752	− 0.02 (− 0.08 to 0.04)	4 (3.8)
*Restriction of living places*							
Staying in one’s own house	26 (25.0)	78 (75.0)	13 (12.5)	91 (87.5)	0.029	0.11(0.02–0.19)	17 (16.3)
Going to public spaces	31 (29.8)	73 (70.2)	15 (14.4)	89 (85.6)	0.001	0.15 (0.07–0.23)	18 (17.3)
Using a bedroom	33 (31.7)	71 (68.3)	18 (17.3)	86 (82.7)	0.002	0.14 (0.06–0.23)	19 (18.3)
Going to other houses	40 (38.5)	64 (61.5)	26 (25.0)	78 (75.0)	0.004	0.13 (0.05–0.21)	17 (16.3)
*Restriction of sanitary behaviours*							
Showering	16 (15.4)	88 (84.6)	16 (15.4)	88 (84.6)	1.000	0.00 (-0.07–0.07)	6 (5.8)
Using used cloth as a napkin	14 (13.5)	90 (86.5)	7 (6.7)	97 (93.3)	0.096	0.07 (0.00–0.13)	10 (9.6)

^a^High frequency; scores of 4 and 5, ^b^Low frequency; scores of 1, 2 and 3, ^c^McNemar’s test, ^d^PD (95% CI); Percent difference (95% confidence interval)

To examine why following traditional menstruation practices became less frequent after moving to Japan, we analysed the responses of women who followed each traditional practice at a high frequency in Nepal. The numbers and percentages of women who belonged to the high-frequency group in Nepal and low-frequency group in Japan, which we termed the ‘reduced frequency group’, are shown in the last column in Table 2. For traditional practices, where more than 20% of the women were in the reduced frequency group (see the last column of Table 2), the relationships between the frequencies of following the practices while the women lived in Japan and their education levels and castes were examined (Table 3). No relationships were observed between the frequencies of traditional practices and education level or caste, except between ‘Contact with male family members’ and education level (*p* = 0.043).

**Table 3:** Traditional menstruation practices in Japan: Caste and education level

Traditional menstruation practices (n^a^)	Education level^b^			Caste^c^		
	High	Low	*p*^d^	High	Low	*p*^d^
*Frequency in Japan*						
Attending a wedding ceremony (n = 46)						
Low (n = 29)	12 (54.5%)	10 (45.5%)	0.581	17 (77.3%)	5 (22.7%)	0.433
High (n = 17)	8 (57.1%)	6 (42.9%)		12 (85.7%)	2 (14.3%)	
Attending to male family members (n = 39)						
Low (n = 27)	13 (48.1%)	14 (51.9%)	0.594	24 (88.9%)	3 (11.1%)	0.494
High (n = 12)	6 (50.0%)	6 (50.0%)		10 (83.3%)	2 (16.7%)	
Contact with male family members (n = 34)						
Low (n = 23)	8 (34.8%)	15 (65.2%)	0.043	20 (87.0%)	3 (13.0%)	0.529
High (n = 11)	8 (72.7%)	3 (27.3%)		9 (81.8%)	2 (18.2%)	
Contact with others (n = 27)						
Low (n = 21)	10 (47.6%)	11 (16.7%)	0.139	19 (90.5%)	2 (9.5%)	0.545
High (n = 6)	5 (83.3%)	1 (16.7%)		5 (83.3%)	1 (16.7%)	
Using the kitchen (n = 47)						
Low (n = 30)	20 (66.7%)	10 (33.3%)	0.157	22 (73.3%)	8 (26.7%)	0.549
High (n = 17)	8 (47.1%)	9 (52.9%)		12 (70.6%)	5 (29.4%)	
Cooking (n = 46)						
Low (n = 29)	20 (69.0%)	9 (31.0%)	0.062	22 (75.9%)	7 (24.1%)	0.450
High (n = 17)	7 (41.2%)	10 (58.8%)		14 (82.4%)	3 (17.6%)	
Using cookware (n = 38)						
Low (n = 24)	14 (58.3%)	10 (41.7%)	0.435	18 (75.0%)	6 (25.0%)	0.365
High (n = 14)	7 (50.0%)	7 (50.0%)		12 (85.7%)	2 (14.3%)	
Touching fruit and fruit trees (n = 40)						
Low (n = 25)	17 (68.0%)	8 (32.0%)	0.276	21 (84.0%)	4 (16.0%)	0.336
High (n = 15)	8 (53.3%)	7 (46.7%)		11 (73.3%)	4 (26.7%)	

^a^Number in the high frequency group in Japan, ^b^High, high school or higher education; Low; secondary school or lower education, ^c^High, Brahmin and Chettri; Low, Others, ^d^Fisher’s exact test

We summarised the responses on difficulties or differences in menstrual practices between Nepal and Japan (Table 4). The background of the women who commented (caste, age, place of origin, and length of stay in Japan) is provided next to their comments. We obtained 22 responses and distributed them among three attitudes toward traditional menstruation practices: negative feelings about traditional menstruation practices, stress due to difficulty in implementing menstruation traditional practices, and social adaptation through the balance between traditional and modern menstruation practices.

**Table 4:** Summary of comments categorised by attitudes toward traditional menstruation practices (n = 22)

*Attitude 1 Negative feelings about traditional practices towards menstruation*	
Comments (Background: Caste, age, hometown, length of stay in Japan)	
1–1.	In Nepal, I had to perform traditional menstruation practices because I lived with my family. They caused me many difficulties. Now that I’m in Japan, I don’t have to perform these practices, so I can live my life normally during my period. Therefore, there is no problem now. (Brahmin, 28, Kathmandu, 0 years)
1–2.	I can spend time during menstruation more comfortably in Japan than in Nepal because I am not forced to follow any traditional menstruation practices in Japan. (Brahmin, 26, Chitwan, 2 years)
1–3.	In Nepal, women performed traditional menstruation practices, which are burdensome. I think that Japanese women are more comfortable during menstruation than Nepalese women. (Brahmin, 41, Kathmandu, 4 years)
1–4.	I wish traditional menstruation practices were abolished in Nepal. (Chettri, 26, Nepal [Others], 6 years)
1–5.	I pay attention to those around me during menstruation in Nepal, but I don’t need to do that in Japan. (Newar, 28, Kathmandu, 6 years)
*Attitude 2 Stress due to difficulty in implementing traditional menstruation practices*	
Comments (Background: Caste, age, hometown, length of stay in Japan)	
2–1.	I performed traditional menstruation practices strictly in Nepal, but I cannot perform them here because of our small living room in Japan. Three women: (Brahmin, 24, Chitwan, 0 years), (Brahmin, 30, Chitwan, 0 years), (Brahmin, 30, Kathmandu, 2 years)
2–2.	I don’t have my own house in Japan, so I have difficulties during menstruation. I want to perform traditional menstruation practices in Japan, but I can’t. (Brahmin, 35, Kathmandu, 8 years)
2–3.	I don’t like to cook meals when guests visit my house during menstruation in Japan. (Brahmin, 29, Lumbini, 1 year)
2–4.	I performed traditional menstruation practices in Nepal without hesitation because I lived with my large family. However, it is difficult for me to perform them in Japan because we have a small family. (Brahmin, 30, Lumbini, 1 year)
2–5.	It is difficult for me to perform traditional menstruation practices (for example, cooking, etc.) in Japan because I live with my family in smaller rooms than in Nepal. (Brahmin, 33, Kathmandu, 1 year)
2–6.	It is harder to perform traditional menstruation practices in Japan than in Nepal. (Brahmin, 42, Kathmandu, 4 years)
*Attitude 3 Social adaptation through the balance between traditional and modern menstruation practices*	
Comments (Background: Caste, age, hometown, length of stay in Japan)	
3–1.	As we had many events for each ethnic group in Nepal, I strictly performed traditional menstruation practices there. Now, I think that the Japanese style of dealing with menstruation is good. (Brahmin, 26, Kathmandu, 0 years)
3–2.	I performed traditional menstruation practices in Nepal and deal with menstruation in the Japanese style now that I live in Japan. Three women: (Brahmin, 27, Lumbini, 0 years), (Brahmin, 30, Nepal [Others], 9 years), (Brahmin, 24, Pokhara, 3 years)
3–3.	I performed traditional menstruation practices in Nepal, but do not perform them in Japan at all. (Brahmin, 24, Pokhara, 3 years)
3–4.	I have no difficulty in dealing with menstruation in the Nepalese or Japanese manner. Three women: (Brahmin, 29, Nepal [Others], 2 years), (Brahmin, 26, Kathmandu, 2 years), (Brahmin, 25, Kathmandu, 2 years)
3–5.	I rarely go to temples during menstruation in Nepal. I do go to temples during menstruation in Japan, but I don’t think it is good. (Brahmin, 38, Kathmandu, 3 years)

## Discussion

In our study, 69.2% of the women had a high education level (high school or higher); this percentage was higher than the net enrolment rate in secondary school (grades 9–12) in Nepal (38.6%) [14]. Further, 67.3% belonged to higher castes (Brahmin and Chettri), which was higher than the percentage in Nepal (28.8%) [15]. Therefore, the Nepalese women who participated in this study had a higher education level and belonged to higher castes than the general population living in Nepal.

The most frequently performed traditional menstruation practices when living in Nepal were restrictions on ‘Going to a temple’ and ‘Attending religious events’ (79.6% and 69.2% in the high-frequency group, respectively) in the ‘Restriction of religious behaviours’ category (Table 2). These percentages in the low-frequency group after moving to Japan were 18.3% for ‘Going to a temple’ and 19.2% for ‘Attending religious events’; these percentages were lower than those for other restrictions (Table 2). This suggests that Nepalese women continued to follow these two restrictive practices even after moving to Japan, although the frequencies decreased. Mukherjee et al. recently reported that most Nepalese women restricted religious behaviours during menstruation, and more than half of Nepalese women believed that they should not visit places of worship or attend religious events during menstruation [4]. One participant in our study said that she rarely went to a temple during menstruation in Nepal, but she went in Japan, although she did not think that it was a good practice (3–5 in Table 4). As there are few Hindu temples in Japan, Nepalese individuals living in Japan go to Japanese temples and shrines that are based on the same polytheism as Hinduism for their spiritual well-being [16]. Nepalese women therefore visited temples during menstruation in Japan against traditional practices, but they generally felt conflicted.

In this study, 14 of 17 traditional menstruation practices were less frequently followed while living in Japan as compared to those while living in Nepal. Based on the data from Nepalese immigrants in Japan [17], the most frequent reasons for living in Japan were studying abroad, staying with family, and technical training. Most Nepalese women living in Japan are 20–35 years old. In this study, the average age of the participants was 29.7 years, and many participants were from single or nuclear families, instead of the traditional three-generation households, common in Nepal. As shown in Table 2, many women in the high-frequency group in Nepal belonged to the low-frequency group after moving to Japan in the categories of ‘Restriction of religious behaviours’, ‘Restriction of contact with others’ and ‘Restriction of cooking and eating’. These three restrictions included practices related to housework, participation in events and communication with family and relatives, suggesting that reduction of traditional practices is caused by changes in the family structure after moving to Japan, consistent with previous studies. Crawford, Menger, and Kaufman reported that many Nepalese women were pressured to continue traditional menstruation practices by their family members, including their mothers-in-law, and concluded that Nepalese women accepted alternative and more modern approaches to menstruation if the prejudice against menstruation was reduced by their families and Nepalese society [3]. Thapa, Bhattarai, and Aro reported that it might be difficult for Nepalese girls and young women to persuade their mothers, mothers-in-law, and other family members to discard traditional practices related to menstruation, even if they understand how to improve their health and hygiene during menstruation [18]. Mukherjee et al. reported that living with a conservative and expanded family was negatively correlated with reduced traditional practices in Nepal [4]. Some comments regarding Attitude 1, as shown in Table 4, supported the idea that traditional practices decreased because the women were not forced to implement the practices by their communities, including family members. We considered that Nepalese women might reduce traditional menstruation practices if they do not live with conservative and expanded families, including their mothers and mothers-in-law.

Recent studies have focused on the relationships between traditional menstruation practices, caste, and education level, and Nepalese women in higher castes have been reported to perform traditional practices in Nepal more frequently than those in lower castes [4, 19]. However, the relationship between traditional practices in Nepal and education level remains controversial. In this study, we examined the relationships between behavioural changes towards traditional practices and caste and education level but found no significant associations, except between education level and restriction of contact with male family members. These results indicated that the attitudes surrounding traditional practices were cultivated through education in the home and primary school, regardless of castes. We consider that early intervention is needed to reduce traditional menstruation practices, regardless of caste or education level.

It is important to note that Nepalese women have ambivalent feelings about traditional menstruation practices. As shown in Attitude 2 in Table 4, Nepalese women were sometimes unable to perform traditional practices because their environment differed from that in Nepal, although they preferred to perform them. In previous studies, Nepalese women living in Nepal commented that they accepted and performed traditional menstruation practices out of respect for the elderly and tradition [3], and cooking restrictions during menstruation provided rest [4]. Thus, it was inferred that some women felt spiritual and physical peace of mind as a positive aspect of menstrual practices. Therefore, some Nepalese women may have positive attitudes regarding the traditional menstruation practices. Future interventions to reduce traditional menstruation practices should not interfere with the well-being of Nepalese women by taking into account their positive attitudes.

We considered ways to reduce these traditional practices in Nepal. Several studies have been conducted on the relationship between traditional practices and menstrual hygiene management (MHM) education in primary schools [10, 11, 20, 21]. MHM was defined as ‘women and adolescent girls using clean menstrual management material to absorb or collect menstrual blood, which can be changed in privacy as often as necessary for the duration of a menstrual period, using soap and water for washing the body as required, and having access to safe and convenient facilities to dispose of used menstrual management materials’ [22]. In low- or middle-income countries around Nepal, MHM education before the first menstruation or during adolescence reduced traditional menstruation practices [23–25]. In Bangladesh, which has similar traditional menstruation practices as Nepal, the usefulness of school-based MHM education was examined in a randomised intervention trial, revealing that girls and their parents reduced traditional practices, such as not touching others and not going to school during menstruation [26]. In our study, most Nepalese women reduced traditional menstrual practices after moving to Japan because of lesser pressure from their family members, especially their mothers or mothers-in-law. Nepalese mothers generally teach their daughters about managing menstruation and pressure them to implement traditional practices [3, 4]. Moreover, no association was found between the traditional menstruation practices and education level, suggesting that the attitudes toward traditional menstruation practices might be acquired at home or in primary or secondary school.

This study proved that the traditional menstruation practices related to housework can be reduced by changes in living conditions, such as the nuclear family associated with migration. However, religious traditional practices did not diminish; in fact, Nepalese women were uncomfortable with diminishing of religious practices during menstruation. The character of menstrual attitudes held by Nepalese women is not uniform, and it was considered important to focus only on the traditional menstruation practices that are detrimental to women's health and to encourage improvement of them by healthcare workers in the community and school settings.

### Implications for clinical practice for nurses caring

We propose implications for the clinical practice of nurses caring to reduce traditional menstrual practices among Nepalese women (Table 5) [27].

**Table 5:** Implications for clinical practice for nursing caring

Promoting menstrual hygiene management (MHM) is a shortcut to eliminating prejudices, misunderstandings, and unhealthy behaviors surrounding traditional menstruation practices
To promote MHM, it is necessary to prepare a supportive environment for girls and women
To eliminate discrimination against menstruation and reduce the enforcement of traditional menstrual practices in Nepal, it is necessary to enact ordinances on reproductive health rights and MHM related to traditional menstruation practices
As a public policy in the school education and health section, it is necessary to train specialists (teachers and health workers) to establish positive social norms
It is necessary for schools and health posts to play a central role in promoting MHM education, including the rights of girls and women to participate in decision-making and in obtaining information

### Limitations

This study had several limitations. First, participants in this study were Nepalese women with high educational backgrounds and castes, and therefore, our results cannot be generalised to all Nepalese women. Second, spouses were not investigated in this study. It is highly likely that the characteristics of spouses influence the behaviour change of Nepalese women. Third, snowball sampling may have resulted in response bias; however, it was effective in sampling a small number of participants staying abroad. Fourth, this study was a retrospective survey of the frequency of implementation of traditional practices towards menstruation in Nepal. The frequencies of menstrual practices in Nepal could be subject to recall bias. For future studies, it would be useful to investigate the traditional practices towards menstruation of Nepalese women living in countries other than Japan and to include women with lower education levels and castes.

## Conclusions

This study is the first to investigate the traditional menstruation practices of Nepalese women living in countries other than Nepal, and resulted in following conclusions: (1) the frequencies of performing most traditional menstruation practices decreased after the women moved to Japan, (2) restrictions related to religious behaviours were still performed frequently while living in Japan, and (3) among women who frequently implemented traditional menstruation practices in Nepal, their educational levels and castes were not significantly related to behavioural changes in traditional practices after moving to Japan.

This study suggests that the attitudes surrounding the traditional menstruation practices change in response to living circumstances. It is necessary to examine in the future what factors of living environment and background are related to behavioural changes for such traditional practices.

## Data Availability

The datasets used and/or analysed in this study are available from the corresponding author on reasonable request. This is to protect the privacy of individual participants. The study subject is sensitive, and it may be possible to identify a person’s beliefs and experiences regarding menstrual behaviour.

## Abbreviation

MHM: Menstrual hygiene management
